# Quantifying the Effects of Visual Road Information on Drivers’ Speed Choices to Promote Self-Explaining Roads

**DOI:** 10.3390/ijerph17072437

**Published:** 2020-04-03

**Authors:** Yuting Qin, Yuren Chen, Kunhui Lin

**Affiliations:** 1 The Key Laboratory of Road and Traffic Engineering, Ministry of Education, Shanghai 201804, China; 1833257@tongji.edu.cn (Y.Q.); 1731276@tongji.edu.cn (K.L.); 2 Shanghai Institute of Intelligent Science and Technology, Tongji University, Shanghai 201804, China

**Keywords:** road characteristics, speed choice, self-explaining road, random forest, convolutional neural network

## Abstract

Roads should deliver appropriate information to drivers and thus induce safer driving behavior. This concept is also known as “self-explaining roads” (SERs). Previous studies have demonstrated that understanding how road characteristics affect drivers’ speed choices is the key to SERs. Thus, in order to reduce traffic casualties via engineering methods, this study aimed to establish a speed decision model based on visual road information and to propose an innovative method of SER design. It was assumed that driving speed is determined by road geometry and modified by the environment. Lane fitting and image semantic segmentation techniques were used to extract road features. Field experiments were conducted in Tibet, China, and 1375 typical road scenarios were picked out. By controlling variables, the driving speed stimulated by each piece of information was evaluated. Prediction models for geometry-determined speed and environment-modified speed were built using the random forest algorithm and convolutional neural network. Results showed that the curvature of the right boundary in “near scene” and “middle scene”, and the density of roadside greenery and residences play an important role in regulating driving speed. The findings of this research could provide qualitative and quantitative suggestions for the optimization of road design that would guide drivers to choose more reasonable driving speeds.

## 1. Introduction

Speed is a crucial factor affected the occurrence and consequences of road traffic crashes [1,2]. Therefore, great importance should be assigned to speed management. There is no doubt that speed reduction measures such as speed limits and red pavement can help to ensure traffic safety. However, in many cases, when drivers fail to perceive risks and develop an inappropriate speed choice, they will "turn a blind eye" to these safeguards [3,4]. Thus, optimizing the layout of roads to improve drivers’ risk perception and guide them towards adjusting their driving behaviors spontaneously is of great significance. When drivers’ expectations coincide with the actual situation, driving will be much safer. The concept of road design based on human factors was put forward by Dutch scholar Theeuwes as early as 1995 [5], and is called self-explaining roads (SERs).

According to the international literature, clarifying the function and classification of roads is an effective measure that can be used to create SERs. This kind of approach focuses on psychological modeling of drivers’ subjective perceptions of roads. The assumption is that drivers usually make judgments unconsciously, based on personality characteristics, existing knowledge, and driving experience [6,7,8,9]. Identifying the road features that drivers use to make discriminations is the key to designing a uniform and predictable road system. Some explanations have been suggested as mechanisms of drivers’ speed choice: (1) Abstract feelings like monotony, physical comfort, task difficulty, and safety are macro factors that determine drivers’ subjective categorization of roads, among which comfort seems to be the most important [10,11]. (2) By visually inspecting clustered road scenarios, road width, lane markings, speed limits, and landscape as discriminant clues. A specific combination of these clues can successfully categorize roads in a way that relates to drivers’ perceptions [12]. It is worth noting that road alignment, which has a great correlation with driving speed on rural roads, is much less important than land use for urban roads. Self-explaining roads have been constructed in New Zealand using these cognitive mechanisms. Observations have shown that by clarifying the classification of roads and expanding the differences between categories, the actual road accident rate is significantly reduced [13,14].

Quantifying the relationships between road characteristics and driving speed is another approach used in the design of SERs. In this approach, vehicle kinematic parameters such as driving speed and lateral deviation are regarded as a reflection of drivers’ thoughts. Research methods used to measure their impact are summarized as follows: (1) Exploring differences in driving behavior under changeable road scenarios through simulated driving experiments [15,16]. By adjusting the target of the experiment, the influence and effectiveness of a specific road feature can be evaluated. (2) Establishing a behavior prediction model and assessing the importance of the predictors [17,18]. According to research findings worldwide, road attributes influencing driving speed can be enumerated: road alignment [19], roadside conditions [16], lane and shoulder width [20], speed limit [21], recovery-zone width, and junction density [22]. In China, operating speed models have been written into the specifications for highway safety audits to evaluate the consistency of road design. However, the models contain only geometric parameters such as horizontal radius and longitudinal slope.

In general, understanding the impact of road features on drivers’ speed choices is the common theme of the above-mentioned research, and it is the key to SERs. Psychological research starts with the general layout of roads and focuses on the information that can be used to discriminate drivers’ subjective judgments. However, research results are usually inferential and vague, and it is hard to generalize universally applicable engineering principles from that. In comparison, the quantitative method seems to be a better choice, but models developed to date vary greatly. Aside from the concept of methodology, the neglect of drivers’ perceptions is another important factor. Although the studies mentioned above have some practical significance for promoting SERs, most of them include “improvement suggestions” but not “design”. Extracting visual road information from images and calibrating its impact on driving speed were the objectives of this paper. Road images from the driver’s visual perspective were separated into layers based on the image semantic segmentation technique. The influences of each layer on driving speed were quantified using speed tags rather than regression coefficients. This is an innovative and practical method of SER design. We hope that the research results can provide suggestions for road layout optimization using speed tags. When a road design needs to be adjusted, the appropriate road facilities can be indexed according to the expected speed tag.

The remaining parts of this paper are organized as follows. The next section introduces the source of the data and the methods of visual road information extraction. The main methodologies of this research are then introduced, including the method used to calculate the speed tag corresponding to each piece of information, the random forest algorithm, and the convolutional neural network. In the following part, speed prediction models were established using these two machine learning algorithms, and the importance of predictors was calculated. The last two sections provide a discussion of the research results and conclude with the contributions.

## 2. Experiments and Data

### 2.1. Naturalistic Driving Experiment 

Naturalistic driving experiments were conducted on five two-lane rural roads in Tibet Province of China. Ten drivers (eight males and two females) took part in the experiment. Their ages ranged from 23 to 50 (mean = 32.9, std = 7.1). All of them had more than three years’ driving experience. A driving recorder (GARMIN GDR35) was fixed on the windshield to obtain videos from the driver’s visual point of view. A three-axis acceleration sensor was synchronized with the camera to gather kinematic vehicle information including driving speed, acceleration, and impulse forces. The total driving mileage was more than 800 km, and over 20 h of driving dash-cam video were obtained from the experiments. Road sections used in our study contained various traffic facilities and roadside landscapes. The traffic volume was relatively low. Given that the response time of most drivers is about 2 s [17], visual road scenarios were matched with driving speed 2 s after. 

### 2.2. Visual Road Information Extraction

Maslow, an American psychologist, established the “hierarchy of needs” theory and studied human behaviors from the perspective of needs [23]. Considering that driving is also a demand-driven behavior, it can be assumed that driving speed is determined by road geometry and modified by the road environment. Road information perceived by drivers can also be described by reference to the hierarchy of requirements. Geometry information is essential and mandatory. In the process of driving, road alignment shows the extension of the road, and the driver has to follow it by controlling the steering wheel and pedal. Although lane markings sometimes do not exist, the driver can subliminally perceive the “shape” of the road and further perform the driving operations [18]. Environment information is additional and optional. For example, for a warning sign to successfully affect driving behavior, it must be detected, understood, and accepted [3]. Therefore, based on the necessity, the visual road information is classified and extracted as follows:

(1) Visual Geometry Information

**Figure 1:**
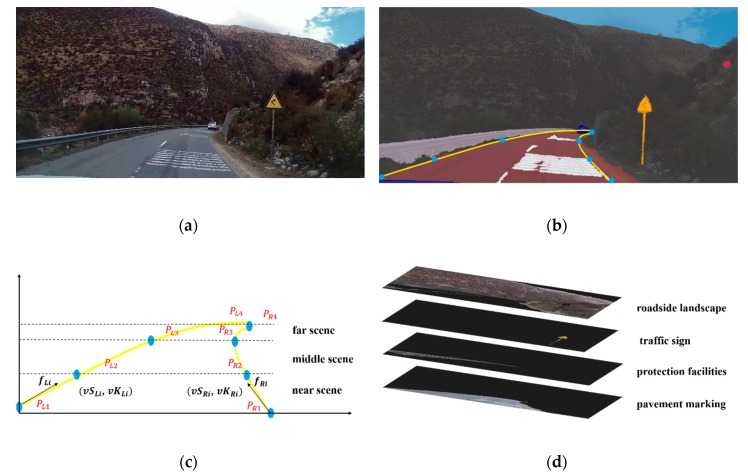
An instance of visual information extraction. (**a**) Original road scenario; (**b**) Lane boundary fitting and semantic segmentation; (**c**) Calculation of shape parameters; (**d**) Extraction of pixels by category.

According to our previous study, the Catmull–Rom spline can fit the road geometry from a driver’s visual perspective well [24,25,26]. Since there is not always a centerline on rural roads, two Catmull–Rom splines were applied to fit the left and right lane boundaries. As shown in Figure 1c, the shape of each spline was controlled by four points ($P_{Li}$,$P_{Ri}$, I = 1,2,3,4) dividing the visual lane into “near scene”, “middle scene”, and “far scene”. Boundary length and average curvature of each region were regarded as the shape parameters, and could describe the visual road geometry. They were calculated as follows.

(1) $${\mathrm{vS}}_{Li(i+1)}{=S}_{Li+1}-S_{Li},$$

(2) $${\mathrm{vK}}_{Li(i+1)}=\frac{f_{Li+1}-f_{Li}}{{\mathrm{vS}}_{Li(i+1)}},$$

(3) $${\mathrm{vS}}_{Ri(i+1)}{=S}_{Ri+1}-S_{Ri},$$

(4) $${\mathrm{vK}}_{Ri(i+1)}=\frac{f_{Ri+1}-f_{Ri}}{{\mathrm{vS}}_{Ri(i+1)}},$$

where i = 1,2,3; ${\mathrm{vS}}_{Li(i+1)}$ is the boundary length between the control point $P_{Li}$ and $P_{Li+1}$ (measured in pixels). $f_{Li+1}$ represents the tangent angle of $P_{Li}$; $vK_{Li(i+1)}$ is the average curvature between $P_{Li}$ and $P_{Li+1}$. ${\mathrm{vS}}_{Ri(i+1)}$, $f_{Ri+1}$, $vK_{Ri(i+1)}$ are parameters for the right lane boundary, which are similar to those for the left one.

(2) Visual Environment Information

Compared with dummy variables, an RGB image, a high-dimensional matrix, can represent richer road environment information. Therefore, road images from the driver’s perspective were used to describe road environment information in this research. Since the disturbance of other vehicles, non-motor vehicles, pedestrians, and livestock was outside the scope of this research, objects observed by drivers were divided into four categories: roadside landscape (e.g., trees, mountains, buildings), traffic signs (e.g., speed limit, sharp curve warning), pavement markings (e.g., red pavement, transverse speed reduction markings), and protection facilities (e.g., guardrail, concrete barrier). An image semantic segmentation technique was used to layer the image. Based on a public dataset named Mapillary Vista, we reproduced the ICnet processed by Zhao et al. (for more information, please see Reference [27]). It performed well on our dataset. Pixels in the original image were classified into four categories and stored in different layers according to their category. Generally, up to four image layers could be separated from one road image. The size of all images was 1920 × 1080. 

The process of visual information extraction is illustrated in Figure 1.

## 3. Methodology

This methodology section consists of three parts: (1) Speed tags were identified by establishing a speed decision model. To calibrate the speed tags of each information, road scenarios were divided into three categories in terms of complexity. (2) The random forest algorithm and a convolutional neural network were used to predict the geometry-determined speed and environment-modified speed. In addition, methods used to measure variable importance in the corresponding models are described.

### 3.1. Identification of Speed Tags

This study hypothesized that speed choice is ultimately determined by geometry information and modified by environment information. A speed decision model was put forward as follows.

(5) $$V=V_{g}+\delta ,$$

(6) $$\delta =\sum_{i}^{n}\omega_{i}\delta_{i},$$

where V is the final speed choice; $V_{g}$ represents for the geometry-determined speed; δ is the general correction caused by environment information; and n is the number of categories of environment information. i = 1,2,3,4, which represent landscape, traffic sign, pavement markings and protection facilities, respectively. $\delta_{i}$ stands for the speed change due to environment information, and $\omega_{i}$ represents its weight. If $\delta_{i}$ is negative, it indicates that the environment information has an inhibiting effect on the driver’s expectation. When $\delta_{i}$ is positive, it means that environment information promotes driver’s expectations.

Road scenarios usually contain a variety of visual information, but there is only one driving speed. Therefore, controlling the variables is the only way to identify the speed tag of each piece of information. Geometry information exists in any road scenario, but the category of environment may include zero, one, or more pieces of information. Depending on the value of n, road scenarios were classified as crude scenarios, single-stimulus scenarios, or multi-stimulus scenarios, in which $V_{g}$, $\delta_{i}$, and $\omega_{i}$ were calibrated. The detailed method used was as follows.

- **In crude scenarios**, roads are laid out in an open field. There are no houses, greenery, or mountains on the side of the road. Traffic facilities do not exist either. Nothing but road geometry affects driving speed. In such road scenarios, $V=V_{g}$, δ=0.
- **In single-stimulus scenarios,** there is one and only one category of environment information. In this case, n=1, $\delta =\delta_{i}$, and $\omega_{i}=1$. By calculating $V_{g}$ and δ, the speed change stimulated by the existing environment information can be estimated.
- **In multi-stimulus scenarios**, there are more than two kinds of environment information. They compete for the driver’s attention and affect driving speed collectively. On the basis of calculations in single-stimulus scenarios, $\delta_{i}$ can be estimated. Statistical methods such as multiple linear regression and the Pearson correlation test were used to investigate $\omega_{i}$.

### 3.2. Machine Learning Algorithm

#### 3.2.1. Random Forest Algorithm

The random forest algorithm was used to build a regression model between the 12 visual shape parameters and $V_{g}$. It is an ensemble algorithm that integrates plenty of single regression trees (CART) by capturing their average of regression as the output. Bagging and boosting techniques are combined in this algorithm. Bagging is a method that can calculate many models at the same time, which can realize parallel computing and improve model robustness. Boosting is an approach for reducing bias. Outliers are highly tolerated, and the importance of explanatory variables can be evaluated in random forest models [28].

The Random Forest Regressor function in scikit-learn (a machine learning toolbox of python) was used to build the model. There are two hyperparameters to be regulated in this model, including the number of the trees in the forest ($n_{trees}$) and the number of variables contained in each split ($n_{features}$). About two-thirds of the samples were used to train the model, and the remaining one-third was used to evaluate the quality of the model [29]. Since driving speed is a continuous variable, MAE (mean absolute error), MSE (mean square error), and $R^{2}$ (explained variance score) were adopted to assess the goodness-of-fit of the model. The grid research and cross-validation methods were utilized to find the optimal combination of the two hyperparameters [30]. The training process can be elaborated as follows.

- The number of trees in the forest was set to $n_{trees}$.
- A subset of the predictors was randomly selected as candidates for splitting, and the sampling size was equal to $n_{features}$.
- The best variable and split-point were picked out among the selected predictors, and each node was split into two subnodes.
- The output of every single tree was aggregated as the final output of the model.

With a pre-trained model, by adding random noise to a certain variable, the reduction of the predicting accuracy can be utilized to measure the relative importance of the predictors [31]. 

#### 3.2.2. Convolutional Neural Network

A convolutional neural network (CNN) was trained to explore the relationship between $\delta_{i}$ and layered images. CNNs are feedforward neural networks. They have exhibited excellent performance on image understanding due to their intelligent way of extracting critical features. Convolution and pooling are two important computing modules in most CNN models. The convolutional layers are designed to extract image features. The main function of pooling is down-sampling, which can remove redundant information. The bottom of a CNN is usually the fully connected (FC) layer. It can map the acquired image features to the outputs [32]. 

There are numerous popular CNN topologies, such as AlexNet [33], GoogleNet, and ResNet [34]. These models perform well in detail extraction, and additional training of these models for new images can save time and produce satisfying results [35]. As the pixels were filtered through semantic segmentation technique, a CNN with a relatively simple topology was developed for use in this study. According to our experience and trial calculation, a convolutional neural network of 10 layers was constructed. It consisted of four convolutional layers, four max pooling layers, and two fully connected layers. A rectified linear unit (ReLU) was inserted after each convolutional layer for non-linear activation. The architecture of the network is shown in Table 1.

To reduce the amount of computation required, images were resized from 1920 × 1080 × 3 to 150 × 150 × 3 before being entered into the network. Additionally, data augmentation techniques were utilized to improve the prediction accuracy and avoid overfitting; segmented images were randomly rotated (range from −10° to 10°), translated (within 20%), scaled (within 20%), and then inputted into the network. The goal of training was to minimize the MSE. A total 20% of the samples were randomly selected as the verification set, and the remaining 80% were considered the training set. The maximum training epoch was 500.

Neural networks perform well to solve non-linear problems, but the model is poorly interpretable. To observe the operation mechanism of the network, a class activation map (CAM) was proposed to visualize the calculation basis of the network. A CAM is a kind of heatmap that can visualize the points used by the model to make a particular decision by highlighting the determinative pixels. The importance value of each pixel can be calculated by multiplying the global average value of the gradients and feature maps obtained from the last convolution layer [36]. After the CNN model was trained, CAMs were drawn to visualize the important environmental information affecting the driving speed.

## 4. Results

### 4.1. Geometry-Determined Speed and Prediction Model

A total of 566 crude scenarios were picked out from the naturalistic driving experiment. The distribution of shape parameters and $V_{g}$ values is shown in Table 2. From this, 378 samples were randomly selected to train the random forest regression model. The input of the model was the 12 visual shape parameters, and the output was $V_{g}$. In the process of grid researching, the MAE was found to decrease initially with increasing $n_{trees}$, and to level off when the value of $n_{trees}$ exceeded 192. In most cases, with the same number of $n_{features}$, MAE was the minimum when $n_{features}$ = 8. Therefore, $n_{trees}$ was set to 192 and $n_{features}$ was equal to 8. The prediction results are illustrated in Table 3. The final MAE was 1.29 and $R^{2}$ was 0.96.

**Figure 2:**
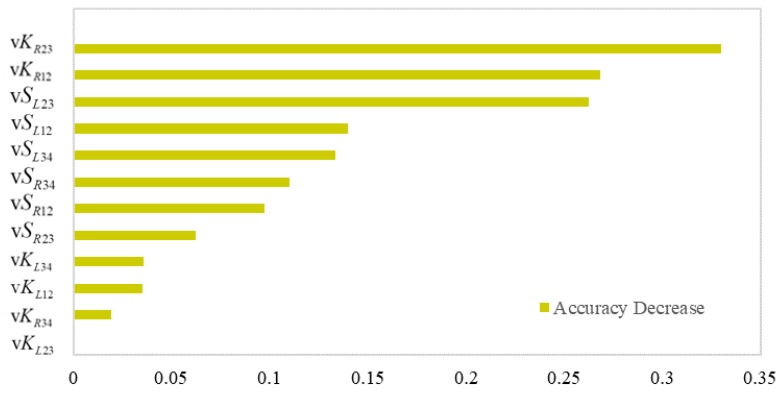
Variable importance in random forest.

Variable importance was calculated and is illustrated in Figure 2. Curvatures of the right boundary in the “middle scene” and the “near scene” were the top-ranked two, with accuracy decreases of 33% and 26.82%. The following is the visual curve length of the left boundary. Interestingly, the curve length of the left boundary was more important than that of the right side. The curvature of the left boundary had less effect on driving speed. When noises were introduced into them, there was less than 4% reduction in prediction accuracy. The average curvature of the right boundary in the “far scene” was an unimportant predictor.

### 4.2. Environment-Modified Speed and Prediction Model

A total of 623 single-stimulus road scenarios were found in the video data. According to the pre-trained geometry-determined speed model, the $V_{g}$ of each scenario was computed. Statistical descriptions of V, $V_{g}$, and $\delta_{i}$ are shown in Table 4. V was generally smaller than $V_{g}$ in single-stimulus scenarios, which indicates that environment information usually inhibited driving speed. Speed change caused by the landscape was relatively large and discrete. It was distributed within the range of (−50 km/h, 10 km/h), and two peaks appeared at −35 km/h and −5 km/h. It was inferred that there were two distinct kinds of landscapes, one with a large negative impact and one with a small negative impact on driving speed. The distributions of $\delta_{i}$ caused by traffic signs and protection facilities were close to normal distributions, and the mean values of them were around −11 km/h and −14 km/h. The $\delta_{i}$ caused by pavement markings was the smallest, while the standard deviation (std.) was the largest.

**Figure 3:**
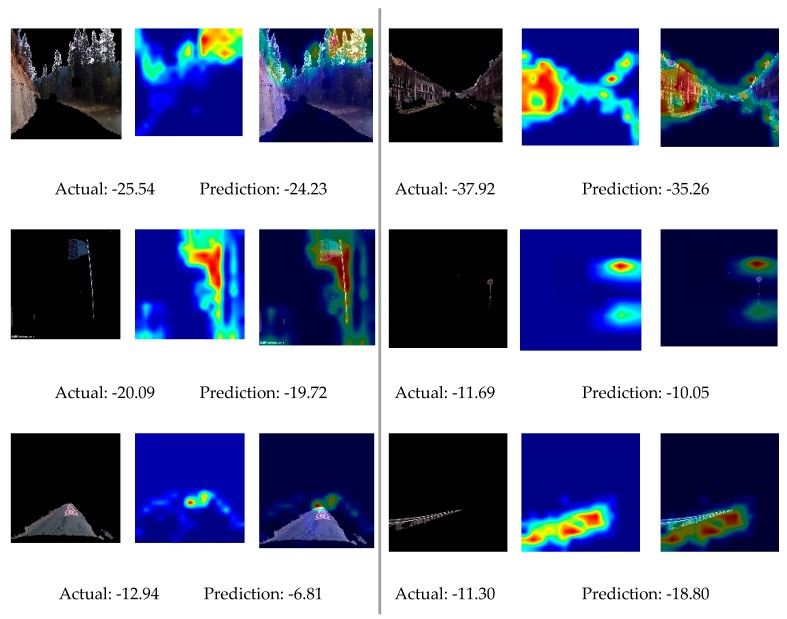
Recognition results from the CNN model.

A convolutional neural network of 10 layers was constructed and trained with the layered image as the input and $\delta_{i}$ as the output. The prediction result is shown in Table 5. It seemed that the CNN model learned to understand the environment images. To analysis the effectiveness of this model, CAMs were plotted and observed. Some examples, including the layered image, the CAM, and their superimposition, are presented in Figure 3. The key information needed to identify the value of $\delta_{i}$ was concluded to be “treetop”, “windows”, and “position of the facility”.

### 4.3. Analysis of the Interaction of Environment Information

A total of 416 multi-stimulus scenarios were extracted from the video data. $V_{g}$ and δ were calculated first. By inputting layered images into the CNN model, speed changes caused by the landscape, traffic sign, lane markings and protection facilities (if present) were calculated and denoted as $\delta_{i}$ (i = 1,2,3,4). Multiple linear regression was performed to evaluate the relationship between δ and $\delta_{i}$. The significance level was chosen to be 0.001, and the result is illustrated in Table 6. The model passed the F test (F (4403) = 9.471) p < 0.001), which indicated that the linear equation was statistically significant. However, only the $\delta_{i}$ caused by landscape and pavement markings had a significant correlation with δ. Additionally, the values of R and $R^{2}$ were quite small, indicating that the goodness of fit and the variance interpretation ability of the model were not satisfactory. Therefore, the interaction of environment information was not a simple linear superposition, nor was $\omega_{i}$ a fixed value.

The deviation between δ and $\delta_{i}$ can be used to roughly measure the relative importance of each piece of information because δ is equal to $\delta_{i}$ when there is no disturbance from other information. The greater the difference, the lower the relative importance. To investigate whether the type of environment information (represented by velocity correction capability $\delta_{i}$) influenced the relative importance, the Pearson correlation coefficient between $\delta_{i}$ and $\left|\delta -\delta_{i}\right|$ was calculated. The results are shown in Table 7. The speed change caused by landscape had significant associations with the relative importance of all categories of environment information. The correlation was mostly positive. The $\delta_{i}$ caused by traffic signs had a significant and negative impact on the importance of landscape and itself, while the effect was positive for pavement markings. The $\delta_{i}$ of pavement markings was positively associated with the effectiveness of the landscape, while the $\delta_{i}$ of protection facilities was significantly and negatively related to the relative importance of itself.

## 5. Discussion 

Previous studies have demonstrated the importance of drivers’ visual perceptions [37], and some scholars have tried to extract color and shape information from road images [38] in order to interpret driving behaviors. In this research, road images were successfully mapped to driving speed based on machine learning methods. These results indicate that extracting visual road information from RGB images can effectively predict driving speed and reveal behavior mechanisms.

According to the random forest model, the curvatures of the right boundary in “near scene” and “middle scene” were the most important factors for drivers’ speed choice. Similar conclusions were reported by Chen et al. [39] after analyzing data on drivers’ eye movements on curved sections. The length of the left lane boundary, which reflects the sight distance to some extent, was also an important factor for driving speed. It is worth noting that the length of the boundary of the left lane was much more important than that of the right side. On curved sections, the visible distance of the left and right lanes is usually different due to perspective transformation. Since the conflict with vehicles traveling in the opposite direction is an important source of risk when driving on two-lane rural roads, the longer the sight distance of the opposite lane, the stronger the driver’s sense of safety. 

As for environment information, the landscape had the greatest influence on the drivers’ speed. “Treetops” and “windows” were found to be crucial information, which reflected the density of roadside vegetation and residence. Charlton et al. [12] demonstrated that roads with abundant greenery and buildings were judged as a destination rather than a direct through-road, and expected speed to be reduced correspondingly. Yu et al. [18] also confirmed that the presence of trees and houses could reduce the probability of speeding. For pavement markings, their location is important, since it takes some time for drivers to react, make decisions, and execute the decisions once they have received such “pulse information”. 

It was also confirmed that the interaction of environment information cannot be superimposed linearly. The change of one type of information will affect the relative importance of other kinds of information. All categories of information have a significant linear relationship with the type of landscape. Landscape with a slight modification on speed has a high tolerance. In such a case, drivers tend to drive freely, and the relative importance of other information is reduced. As for traffic signs, those with little impact on speed are usually information delivery or danger warning signs, such as “villages ahead”, “fast curves ahead”, etc. Compared with mandatory signs, these suggestive signs make drivers more aware of the road conditions, so the effectiveness of landscape information is improved.

## 6. Conclusions

This study aimed to quantify the influence of visual road information on driving speed, hoping to inform safer speed choices by optimizing road design. There have been many studies on driving speed. However, drivers’ perceptions of the road, as well as engineering practicality, are often ignored. In this study, visual road information was categorized as visual geometry information and visual environment information, which were extracted from RGB images from the driver’s perspective. Prediction models for geometry-determined speed and environment-modified speed were established based on the random forest algorithm and a convolutional neural network. 

Moreover, the importance of information was also computed. The analysis showed that curvatures of the right boundary in the “middle scene” and the “near scene” were critical factors influencing drivers’ speed choice. Landscape can affect driving speed dramatically. It was inferred that there would be two distinct landscapes that could reduce the speed by about 35/h and 5 km/h. Speed changes caused by traffic signs and protection were relatively small. In multi-stimulus scenarios, the effectiveness of these road facilities is often influenced by the category of the landscape.

Compared with previous studies, this study was more concerned with the one-to-one correspondence between specific information and speed. The logic of the study complied with the process of road design; that is, the geometry of the road is selected first, and the landscape and facilities are arranged subsequently. With speed tags as the basis of design, drivers’ needs can be better fitted.

One of the limitations of this study was that the models only analyzed driving speeds on rural roads in China; the fitness of the model for other kinds of roads should be further tested. In future study, large-scale field tests could be implemented to obtain more comprehensive driver behavior data. It should be pointed out that the study contributed to road image understanding from the driver’s perspective, and proposed an entirely new idea for the design of SER. With the development of intelligent algorithms, it will be possible to realize intelligent optimization of road layout based on driving demand in the future.

## Figures and Tables

**Table 1:** The architecture of the convolutional neural network.

Layer	Output Size	Number of Parameters
conv3-32, ReLU	(14814832)	896
2×2 max pool	(747432)	0
conv3-64, ReLU	(727264)	18496
2×2 max pool	(363664)	0
conv3-128, ReLU	(3434128)	73856
2×2 max pool	(1717128)	0
conv3-128, ReLU	(1515128)	147584
2×2 max pool	(77128)	0
FC-512, ReLU	(1512)	3211776
FC-3	(11)	513

**Table 2:** Distribution of shape parameters and geometry-determined speed.

Parameter	Minimum	Maximum	Mean	Standard Deviation
$vK_{L12}$	−8.91 × 10^−5^	1.29 × 10^−3^	8.89 × 10^−5^	1.74 × 10^−4^
$vK_{L23}$	−2.02 × 10^−2^	8.40 × 10^−3^	−2.95 × 10^−4^	4.02 × 10^−3^
$vK_{L34}$	−3.60 × 10^−2^	1.05 × 10^−2^	−4.19 × 10^−3^	8.17 × 10^−3^
$vS_{L12}$	177.51	652.13	453.71	102.16
$vS_{L23}$	78.62	566.34	312.12	100.40
$vS_{L34}$	56.26	592.78	218.71	123.33
$vK_{R12}$	−1.85 × 10^−3^	4.50 × 10^−4^	−1.04 × 10^−4^	2.63 × 10^−4^
$vK_{R23}$	−7.08 × 10^−3^	2.74 × 10^−2^	3.14 × 10^−3^	6.69 × 10^−3^
$vK_{R34}$	−1.57 × 10^−2^	3.40 × 10^−2^	1.05 × 10^−3^	7.42 × 10^−3^
$vS_{R12}$	136.19	697.37	338.27	105.56
$vS_{R23}$	82.86	460.32	232.54	83.85
$vS_{R34}$	38.27	529.91	204.54	101.29
$V_{g}$	65.00	111.00	81.64	9.41

**Table 3:** Prediction result of random forest.

Training Set			Testing Set		
Sample Size	MAE	$R^{2}$	Sample Size	MAE	$R^{2}$
378	2.01	0.91	188	1.29	0.96

**Table 4:** The statistical descriptions of actual speed, geometry-determined speed and environment-modified speed.

Semantic Information Category	V		$V_{g}$		δ $\left(\delta_{i}\right)$		Distribution of δ $\left(\delta_{i}\right)$
	Mean	Std.	Mean	Std.	Mean	Std.	
Landscape	66.3	12.41	87.79	6.84	−26.23	11.26	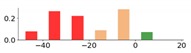
Traffic sign	75.69	11.83	87.86	4.96	−11.35	12.1	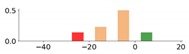
Pavement marking	77.93	10.4	88.18	4.73	−9.7	12.63	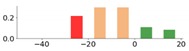
Protection facility	70.17	9.18	84.77	8.45	−13.8	9.13	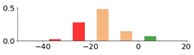

**Table 5:** Training result of the convolutional neural network (CNN).

Training Set			Testing Set		
Sample Size	MSE	MAE	Sample Size	MSE	MAE
450	55.09	6.89	173	37.37	4.58

**Table 6:** Results of multiple linear regression.

**ANOVA**					
**Model**	**Sum of Squares**	**df**	**Mean Square**	**F**	**Sig.**
Regression	4720.78	4	1180.19	9.47	<0.001
Residual	50,217.38	403	124.61		
Total	54,938.16	407			
**Model Summary**					
R	$R^{2}$	Adjusted $R^{2}$		Std. Error of the Estimate	
0.293	0.086	0.076856		11.16283	
**Coefficients**					
Variables	Unstandardized Coefficients		Standardized Coefficients	t	Sig.
	B	Std. Error	Beta		
(Constant)	−12.852	2		−6.427	<0.001
Landscape	0.224	0.054	0.209	4.128	<0.001
Traffic sign	0.092	0.089	0.058	1.039	0.299
Pavement marking	0.554	0.147	0.197	3.763	<0.001
Protection facilities	−0.067	0.076	−0.049	−0.887	0.375

**Table 7:** Result of Pearson correlation analysis.

			Relative Importance			
			Roadside Landscape	Traffic Sign	Pavement Markings	Protection Facilities
**Category**	Roadside landscape	Pearson correlation	−0.450 ** ^1^	0.305 **	0.248 **	0.356 **
		Sig. (2-tailed)	0.000	0.001	0.008	0.000
	Traffic Signs	Pearson correlation	−0.323 **	−0.313 **	0.514 **	0.001
		Sig. (2-tailed)	0.000	0.000	0.009	0.994
	Pavement Markings	Pearson correlation	0.332 **	0.379	0.089	0.156
		Sig. (2-tailed)	0.000	0.062	0.316	0.227
	Protection Facilities	Pearson correlation	−0.001	−0.017	0.022	−0.555 ^**^
		Sig. (2-tailed)	0.993	0.895	0.864	0.000

^1^ ** represent that the correlation is significant at the 0.01 level (2-tailed).
